# The Strengths and Difficulties Questionnaire Dysregulation Profile Teacher-Report: Psychometric Properties and Associations with Aspects of School Experience

**DOI:** 10.1007/s10578-022-01441-5

**Published:** 2022-10-13

**Authors:** Valentina Levantini, Marina Camodeca, Carmen Gelati

**Affiliations:** 1 Department of Languages and Literatures, Communication, Education and Society, University of Udine, Udine, Italy; 2 Department of Psychology, University of Milano – Bicocca, Milan, Italy

**Keywords:** Dysregulation Profile, SDQ, School Adjustment, Reliability, Confirmatory Factor Analysis

## Abstract

Emotional, cognitive, and behavioral dysregulation is a risk factor for severe outcomes, calling for reliable measures to assess it, including the Strengths and Difficulties Questionnaire Dysregulation Profile (SDQ-DP) defined by 5 or 15 items. This study explored for the first time the factor structure, internal consistency, and test-retest reliability of the SDQ-DPs teacher-report in a sample of Italian children (*N* = 1000; age 7–12 years). The gender invariance of the SDQ-DPs, construct validity, and associations with school variables were also evaluated. A first-order model for the SDQ-DP 5-item and a bifactor model for the SDQ-DP 15-item best fitted the data. Full measurement invariance across gender was confirmed only for the 15-item scale. Internal and test-retest reliabilities were weaker for the 5-item scale. Both SDQ-DPs were similarly associated with a measure of emotion regulation skills and some school variables. This study provides indications for a more conscious use of the two scales.

## Introduction

The Strengths and Difficulties Questionnaire (SDQ) [1] is one of the most widely used behavioral screening measures, and it assesses children’s and adolescents’ difficulties (i.e., Hyperactivity–Inattention, Emotional Symptoms, Conduct Problems, and Peer Problems) and assets (i.e., Prosocial Behavior) from the parents’, teachers’, or youths’ point of views. The SDQ is available in several languages and for a large age span (from early childhood to adulthood), wholly open-access, and substantially shorter than equally valid screening instruments [2]. These aspects, together with the SDQ versatility, contributed to its growing use in research.

Along with the more common use of the SDQ to explore the abovementioned difficulties or internalizing/externalizing problems [3], more recently, some of its items have been employed to assess emotional, cognitive, and behavioral dysregulation in children and adolescents through the so-called Dysregulation Profile (DP). Dysregulation is here intended as the disruption of different aspects of self-regulation [4], including affect (e.g., difficulties in managing emotions), cognition (e.g., attention problems), and behavior (e.g., aggression). Even though the first DP developed was based on the Child-Behavior Checklist (CBCL) [5–7], an SDQ-DP, primarily defined with a 5-item or a 15-item scale, is also available [8, 9]. The SDQ-DP 5-item was first proposed by Holtmann et al. [8], who isolated the combination of SDQ items that best captured signs of dysregulation as described by the CBCL-DP. The items selected belonged to the subscales of Hyperactivity– Inattention (item 2: *“Restless, overactive, cannot stay still”*), Conduct Problems (item 12: “*Often fights with other youth or bullies them”*; item 22: “*Steals from home, school, or elsewhere”*) and Emotional Symptoms (item 8: *“Has many worries or often seems worried”*; item 13: *“Often unhappy, depressed, tearful”*). The SDQ-DP 15-item [9] instead involves all the items of the Hyperactivity– Inattention, Conduct Problems, and Emotional Symptoms subscales, somehow tracing the CBCL-DP structure, which combines the scores of all the items of the Attention Problems, Aggressive Behavior, and Anxiety/Depression scales [5].

The significance of assessing the DP relies on evidence indicating that the co-occurrence of emotional, cognitive, and behavioral dysregulation in children and adolescents leads to severe and burdensome outcomes. Studies have shown that the DP is associated with internalizing and externalizing problems and a heightened risk for psychopathology in clinical and community samples [8, 10, 11]. Moreover, research has suggested that high levels of dysregulation might also impact youths’ psychosocial functioning, relationships with their peers, as well as their school functioning, and adjustment [11–13].

Despite the undeniable importance of reliable measures to identify youths at great risk for dysregulation, studies investigating the SDQ-DP structure and psychometric properties are still meager. In a first study, Holtmann et al. [8] developed and evaluated the psychometric properties of the SDQ-DP 5-item parent-report in a clinical sample of children and adolescents (age 5–17 years). Results showed a rather low internal consistency (Cronbach’s α = 0.52); however, the SDQ-DP appeared to be highly correlated with the CBCL-DP and able to accurately discriminate between children with and without co-occurring emotional, behavioral, and cognitive dysregulation. Subsequently, Deutz et al. [9] extensively explored the properties of both the SDQ-DPs 5-item and 15- item. Findings showed higher internal consistency for the SDQ-DP 15-item with Cronbach’s α ranging from 0.80 to 0.87 across different informants (i.e., parent, teacher, youth) and developmental periods. Both SDQ-DPs, though, were similarly associated with two markers of self-regulation (i.e., ego-resilience and effortful control) and other long-term outcomes (e.g., antisocial behavior). Finally, the authors showed that, for the SDQ-DP 15-item, a bifactor model best fitted the data, also providing evidence of measurement invariance across reporters and time.

More recently, Levantini et al. [10] explored the structure and psychometric properties of the SDQ-DP 5-item parent-report in a community sample of Italian early adolescents, showing that it reliably (α = 0.76) assessed a single construct and was associated with negative outcomes in the school context (i.e., teacher-reported internalizing and externalizing problems). To the best of our knowledge, no previous study investigated the psychometric properties of the Italian version of the SDQ-DP teacher-report.

Validation studies of the Italian version of the SDQ indicated that the teacher version might have greater internal reliability than the parent form [14, 15], suggesting that teachers can be highly sensitive to their students’ emotional, attentional, and behavioral difficulties and provide valuable information about their well-being [16]. In addition, teachers are trained to observe students and compare them on several aspects and have the opportunity to spend much time with them. Increasing our knowledge about the psychometric properties and correlates of the SDQ-DP teacher-report would unveil its potential as a screening tool to identify at-risk students and facilitate the implementation of preventive and targeted interventions in the school context. Consistently, the current study sought to explore for the first time the factor structure, internal consistency, and test-retest reliability of the SDQ-DPs 5-item and 15-item teacher-report in a sample of Italian children. The study also aimed to explore the gender invariance of the SDQ-DPs and to preliminary test their construct validity by exploring their association with a measure of emotion regulation. Finally, we investigated the associations between the SDQ-DPs and different outcomes in the school context. In particular, we expected that the DPs would be positively associated with peer problems and conflict with the teachers, and negatively associated with academic performance, academic self-efficacy, closeness with the teacher, and school climate.

## Method

### Participants and Procedure

Participants involved in the present study were drawn from two projects investigating several variables. The whole sample included 1000 students (51.8% males) aged between 7 and 12 years (mean age = 8.77, *SD* = 0.76). Four hundred (40%) children were 3rd graders, 467 (46.7%) were 4th graders, and the remaining 133 (13.3%) were 5th graders at the moment of the first data collection. The majority of the students (91.10%) were born in Italy. Before filling in the questionnaires, parents and teachers signed a written informed consent; the students also agreed to participate in the study. The procedures were in accordance with the 1964 Helsinki declaration and its later amendments or comparable ethical standards. The two projects were approved by the Ethical Committees of the Italian Universities of Udine and Milano-Bicocca.

## Measures

*Dysregulation Profile.* The Strengths and Difficulties Questionnaire (SDQ) [1] is a 25-item questionnaire that assesses youths’ emotional and behavioral difficulties and strengths. It is available in different formats, including teacher-report. Answers are provided on a 3-point Likert scale (0 = *not true*, 1 = *somewhat true*, 2 = *certainly true*). The SDQ involves five subscales, with five items each: Hyperactivity–Inattention, Emotional Symptoms, Conduct Problems, Peer Problems, and Prosocial Behavior. For the current study, we used the Italian version of the SDQ teacher-report [15] to assess students’ DPs. Teachers completed the SDQ at the baseline (*N* = 1000) and 6 (*N* = 375) or 12 (*N* = 499) months later. The SDQ-DP 5-item has been computed following Holtmann et al.’s [8] definition, while the SDQ-DP 15-item has been computed by summing the scores of the items of the Hyperactivity–Inattention, Emotional Symptoms, and Conduct Problems scales [9].

*Peer problems.* The relative subscale of the SDQ was employed to measure peer problems in the school context (e.g., “*Rather solitary, tends to play alone*”). In the current sample, Cronbach’s α for this scale was 0.67.

*Emotion regulation skills*. The Emotion Regulation Checklist (ERC) [17, 18] is a 24-item teacher-report measure assessing processes central to emotionality and regulation in children. Answers are rated on a 4-point Likert scale (from 1 = *almost always* to 4 = *never*). The ERC involves two subscales: Emotion Regulation (8 items, e.g., “*Can say when she/he feels sad, angry or mad, fearful or afraid*”), which investigates positivity and appropriateness of emotional responses and emotion awareness, and Lability/Negativity (15 items, e.g., “*Is prone to angry outbursts/tantrums easily*”), which indicates dysregulated negative affect, arousal, and mood. In the current sample, Cronbach’s α were 0.73 and 0.88 for the Emotion Regulation and Lability/Negativity subscales, respectively. The ERC was available for 393 students.

*Academic performance.* School success was operationalized as grades obtained in the first term; scores ranged from 0 to 10 (with ten being the best mark). Parents reported the grade obtained by their child in school subjects, which was averaged in order to have one score. School grades were available for 451 students.

*Academic Self-Efficacy.* The Academic Self-Efficacy Scale [19] is a 19-item measure assessing how much children feel academically efficacious (e.g., “*How well can you learn Italian grammar*?”, “*How well can you finish homework assignments by deadlines*?”). Students rate the items on a 5-point Likert scale (from 1 = *absolutely incompetent* to 5 = *absolutely competent*). For this study, we used 13 items of the original scale to assess scholastic self-efficacy. In the current sample, Cronbach’s α was 0.84, and scores were available for 981 students.

*Student-Teacher Relationship.* The Student-Teacher Relationship Scale (STRS) [20] is a teacher-report scale assessing how teachers perceive the relationship with their students. Teachers rate the items on a 5-point Likert scale (from 1 = *definitely does not apply* to 5 = *definitely applies*). For this study, we used 8 items to assess student-teacher Closeness (e.g., “*I share an affectionate, warm relationship with this child*”) and 11 items to assess student-teacher Conflict (e.g., “*This child and I always seem to be struggling with each other*”). In the current sample, Cronbach’s α were 0.88 and 0.89 for the Closeness and Conflict subscales, respectively. The evaluation of the student-teacher relationship was available for 990 students.

*Perception of School Climate.* An adaptation of the Georgia Student Health Survey 2.0 (GSHS) [21, 22] was employed to assess children’s perceptions of school connectedness, peer and adult social support, and cultural acceptance. It consists of 16 items (e.g., “*I feel like I fit in at my school*,” “*Adults in this school treat all students with respect*”) with a 4-point Likert response modality (from 1 = *completely disagree* to 4 = *completely agree*). In the current sample, Cronbach’s α was 0.84. The evaluation of the school climate was available for 572 students.

## Statistical Analyses

**Fig. 1:**
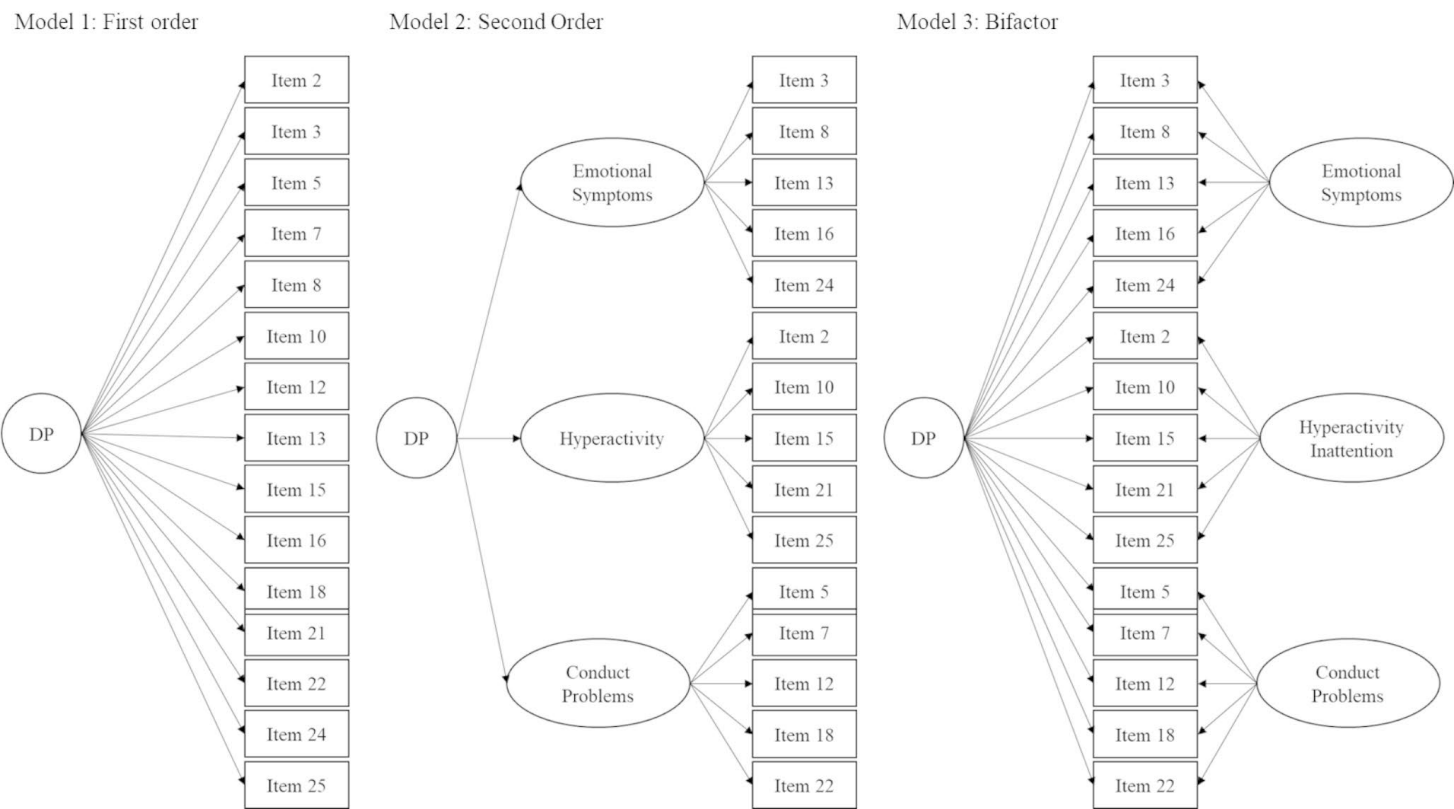
Competing models tested for the SDQ-DP 15-item

As preliminary analyses, we performed descriptive statistics with IBM SPSS Statistics for Windows, Version 26.0. A confirmatory factor analysis (CFA) was conducted to test the best fitting models using IBM SPSS Amos, Version 23.0. The CFA was performed using the maximum likelihood estimator. Specifically, we tested a first-order one-factor model for the SDQ-DP 5-item [10] and three competing models [9] for the SDQ-DP 15-item: Model 1: first-order one-factor model, with all the 15 items loading into a mono-factorial structure; Model 2: a second-order model with the three SDQ subscales (Hyperactivity–Inattention, Emotional Symptoms, Conduct Problems) as first-order constructs and a second-order factor (Dysregulation Profile); Model 3: a bifactor model where all the items loaded into the general factor Dysregulation Profile and, at the same time, into their respective specific factors (Hyperactivity-Inattention, Emotional Symptoms, Conduct Problems) (see Fig. 1).

As recommended, we assessed the model goodness-of-fit using different indices, including chi-square (χ^2^), Comparative Fit Index (CFI), Standardized Root Mean Square Residual (SRMR), and Root Mean Square Error of Approximation (RMSEA). Chi-square is reported but not interpreted as usually influenced by large samples and/or complex models [23]. Values of CFI ≥ 0.90, and SRMR and RMSEA ≤ 0.08 were interpreted as acceptable fit [24–26]. The comparison between competing factorial models for the SDQ-DP 15-item has been analyzed using ∆χ^2^ and Aikane Information Criterion (AIC) and Bayesian Information Criterion (BIC) values.

We used Multiple-Group analysis to test the invariance across gender for the best fitting models at the configural, metric, and scalar levels. We first evaluated the goodness-of-fit of the best fitting models separately for boys and girls. The models were then tested in both gender groups concurrently (configural models). Equal constraints were then imposed on all factor loadings across groups (metric models). The metric models were compared with the configural ones. Finally, all intercepts were constrained to be equal (scalar models), and the scalar models were compared with the metric models. To compare nested models for invariance across gender, we used ΔCFI and ΔRMSEA, with measurement invariance holding if the changes in fit statistics between models were ≤ 0.015 for ΔRMSEA and ≤ 0.01 for ΔCFI [27]. We selected these parameters because they are less sensitive to sample size and more sensitive to a lack of invariance than χ^2^ statistics [28].

The SDQ-DPs internal consistency was assessed with Cronbach’s α, mean inter-item correlations (MIC), and McDonald’s ω. The SDQ-DPs test-retest reliability was assessed by testing the zero-order correlations between the SDQ-DPs assessed at the baseline and 6 or 12 months later. Construct validity and associations with variables in the school context were evaluated with partial correlations, using gender as control variable.

## Results

### Descriptive Statistics

Table 1 shows the descriptive statistics for the whole sample, with gender comparisons.

**Table 1:** Descriptive statistics and gender comparisons

	Whole Sample		Males		Females		
	*mean*	*SD*	*mean*	*SD*	*mean*	*SD*	*t*
SDQ-DP 5-item	1.36	1.66	1.75	1.83	0.93	1.32	8.18***
SDQ-DP 15-item	5.24	5.19	6.35	5.56	4.05	4.49	7.22***
SDQ-DP 5-item 6 months	1.18	1.51	1.44	1.56	0.93	1.41	3.34***
SDQ-DP 15-item 6 months	4.89	4.81	5.78	5.07	4.03	4.39	3.54***
SDQ-DP 5-item 12 months	1.16	1.54	1.42	1.67	0.85	1.31	4.33***
SDQ-DP 15-item 12 months	4.84	5.13	5.75	5.53	3.75	4.38	4.43***
Lability/Negativity	1.60	0.49	1.74	0.54	0.04	5.38	5.39***
Emotion Regulation	3.08	0.50	2.96	0.53	3.18	0.45	-4.43***
Academic Performance	8.88	0.85	8.76	0.82	9.02	0.86	-3.24***
Self-Efficacy	3.61	0.65	3.66	0.63	3.57	0.67	2.20*
Closeness with the teacher	3.94	0.78	3.82	0.79	4.08	0.73	-5.49***
Conflict with the teacher	1.29	0.54	1.38	0.63	1.20	0.42	5.41***
School Climate	3.28	0.45	3.22	0.49	3.35	0.39	-3.69***
Peer Problems	0.22	0.32	0.25	0.33	0.19	0.30	3.36***
*Note. SD*: Standard Deviation; SDQ-DP: Strengths and Difficulties Questionnaire – Dysregulation Profile. Except for the SDQ-DPs, variables have been computed as mean scores. * *p* ≤ .05; *** *p* ≤ .001							

Overall, females reported significantly lower scores on the DP, regardless of the number of items used to assess it and time points. Gender differences also emerged for the other measures employed in the current study, with females reporting significantly higher academic performance, emotion regulation, student-teacher closeness, and school climate scores. Males, instead, reported higher levels of lability/negativity, academic self-efficacy, student-teacher conflicts, and peer problems.

## Confirmatory Factor Analyses

*SDQ-DP 5-item*. The CFA revealed poor goodness-of-fit for the SDQ-DP 5-item (χ^2^(5) = 227.68, *p* ≤ .001; CFI = 0.70; SRMR = 0.106; RMSEA = 0.211). The modification indices (M.I. = 197.01) suggested a strong covariance between the error terms of item 8 (“*Many worries”*) and item 13 (*“Often unhappy, downhearted”*). Both items belong to the Emotional Symptoms subscale of the SDQ and have apparent item content overlap; therefore, we decided to include this error covariance parameter in the model [24, 29]. The addition of this parameter significantly improved the fit indices of the model (χ^2^(5) = 5.09, *p* = .280; CFI = 0.99; SRMR = 0.019; RMSEA = 0.017). Factor loadings (Table 2) varied from 0.18 to 0.89, with the lowest factor loading associated with item 8 (“*Many worries”*) and the highest with item 12 (“*Often fights with other children*”).

**Table 2:** Standardized factor loadings of the SDQ-DP 5 item

Item	DP	90% C.I.
2: Restless, overactive	0.54	0.46 − 0.63
8: Many worries	0.18	0.11 − 0.15
12: Often fights	0.89	0.79 − 0.99
13: Often unhappy	0.29	0.22 − 0.38
22: Steals	0.27	0.19 − 0.34
*Note*. DP: Dysregulation Profile. 90% C.I.: Bias Corrected 90% Confidence Interval		

*SDQ-DP 15-item*. Table 3 shows the fit indices of the competing models for the SDQ-DP 15-item. The CFA revealed that a bifactor model best fitted the data. Small modification indices and their relative expected parameter changes, along with acceptable fit indices, suggested that no further modifications were needed for the bifactor model.

**Table 3:** Fit indices of the competing models for the SDQ-DP 15-item

Model	χ^2^	df	CFI	SRMR	RMSEA	AIC	BIC	∆χ^2^
1) First Order	2028.00***	90	0.68	0.103	0.147	2088.00	2235.18	
2) Second Order	1146.13***	87	0.82	0.071	0.111	1212.13	1374.02	1 vs. 2: ∆χ^2^ = 881.87***
3) Bifactor	593.19***	75	0.91	0.058	0.082	683.19	903.94	2 vs. 3: ∆χ^2^ = 552.94***
*** *p* ≤ .001								

Table 4 shows the standardized factor loadings along with 90% bias-corrected confidence intervals.

**Table 4:** Standardized factor loadings of the SDQ-DP 15-item

Item	DP	Emotional	Hyperactivity	Conduct
3: Headaches	0.26 [0.20 − 0.33]	0.43 [0.36 − 0.51]		
8: Many worries	0.24 [0.17 − 0.30]	0.67 [0.60 − 0.73]		
13: Often unhappy	0.31 [0.24 − 0.38]	0.53 [0.45 – 0.60]		
16: Nervous or clingy	0.44 [0.38 − 0.49]	0.50 [0.43 − 0.56]		
24: Many fears	0.25 [0.18 − 0.32]	0.66 [0.59 − 0.72]		
2: Restless, overactive	0.79 [0.74 − 0.83]		0.33 [0.23 − 0.43]	
10: Fidgeting/ squirming	0.77 [0.71 − 0.81]		0.35 [0.26 − 0.46]	
15: Easily distracted	0.77 [0.73 – 0.81]		- 0.24 [-0.34 − - 0.16]	
21: Thinks things out^R^	0.66 [0.61 − 0.70]		- 0.28 [-0.34 − - 0.19]	
25: Sees tasks through to the end^R^	0.68 [0.62 − 0.74]		- 0.50 [-0.61 −- 0.41]	
5: Loses temper	0.46 [0.39 − 0.53]			0.36 [0.26 − 0.44]
7: Generally obedient^R^	0.65 [0.60 − 0.69]			0.24 [0.17 − 0.32]
12: Often fights	0.61 [0.55 − 0.67]			0.54 [0.44 − 0.63]
18: Lies or cheat	0.54 [0.47 − 0.59]			0.53 [0.44 − 0.61]
22: Steals	0.21 [0.15 − 0.28]			0.29 [0.16 − 0.41]
Note. DP: Dysregulation Profile. 90% bias-corrected confidence intervals are shown in square brackets. R = reverse-coded				

## Multiple-Group Analyses

*SDQ-DP 5-item*. The first-order model (with errors covariance) fit of the SDQ-DP 5-item was acceptable in both males (χ^2^(4) = 5.44, *p* = .248; CFI = 0.99; RMSEA = 0.026) and females (χ^2^(4) = 16.90, *p* = .002; CFI = 0.94; RMSEA = 0.082). The fit indices were acceptable for the configural (χ^2^(8) = 22.34, *p* = .004; CFI = 0.98; RMSEA = 0.042) and metric models (χ^2^(13) = 33.26, *p* = .002; CFI = 0.97; RMSEA = 0.040), with ΔCFI (0.00) and ΔRMSEA (0.002) suggesting full metric invariance.

The scalar model showed acceptable fit indices (χ^2^(17) = 80.74, *p* ≤ .001; CFI = 0.91; RMSEA = 0.061). However, when compared to the metric model, ΔCFI (0.06) and ΔRMSEA (0.021) did not provide evidence of full scalar invariance. We searched for sources of gender non-invariance, and the results showed that the intercepts of item 2 (Males: 0.63; Females: 0.21) and item 12 (Males: 0.47; Females: 0.20) were most likely interfering with scalar invariance. We created a partial scalar model with the intercepts of these items free to vary across the groups. This model showed acceptable fit indices (χ^2^(14) = 33.71, *p* = .002; CFI = 0.97; RMSEA = 0.038). The partial scalar model was compared to the metric one, and ΔCFI (0.00) and ΔRMSEA (0.002) suggested partial scalar invariance.

*SDQ-DP 15-item*. The bifactor model fit was acceptable in both males (χ^2^(75) = 369.96, *p* ≤ .001; CFI = 0.91; RMSEA = 0.087) and females (χ^2^(75) = 16.90, *p* ≤ .001; CFI = 0.90; RMSEA = 0.084), with the RMSEA slightly higher than recommended in both groups.

The model fit indices were acceptable for the configural (χ^2^(150) = 700.31, *p* ≤ .001; CFI = 0.90; RMSEA = 0.061), metric (χ^2^(176) = 798.86, *p* ≤ .001; CFI = 0.89; RMSEA = 0.059), and scalar models (χ^2^(179) = 837.760, *p* ≤ .001; CFI = 0.89; RMSEA = 0.059). The comparison between the configural and metric models (ΔCFI = 0.01, ΔRMSEA = 0.002), and between the metric and scalar models (ΔCFI = 0.00, ΔRMSEA = 0.00) suggested full metric and scalar invariance.

## Internal Consistency and Test-Retest Reliability

The SDQ-DP 5-item had a Cronbach’s α equal to 0.60, MIC value of 0.23, and McDonald’s ω of 0.64. The SDQ-DP 15 item had a Cronbach’s α equal to 0.87, MIC value of 0.31, and McDonald’s ω of 0.88.

At each time point, the SDQ-DPs 5-item and 15-item were significantly and positively correlated with each other (baseline: *r* = .89, *p* ≤ .001; 6 months: *r* = .87, *p* ≤ .001; 12 months: *r* = .87, *p* ≤ .001). The SDQ-DP 5-item at the baseline was positively associated with the SDQ-DP 5-item assessed 6 months (*r* = .62, *p* ≤ .001) or 12 months (*r* = .65, *p* ≤ .001) later. Similarly, the SDQ-DP 15-item at the baseline was positively associated with the SDQ-DP 15-item assessed 6 months (*r* = .75, *p* ≤ .001) or 12 months (*r* = .74, *p* ≤ .001) later.

## Construct Validity and Association with Variables in the School Context

Construct validity has been preliminarily assessed by exploring the association between the SDQ-DPs and the Emotion Regulation Checklist subscales, while controlling for gender. Both SDQ-DPs were significantly and positively associated with lability/negativity (SDQ-DP 5-item: *r* = .62, *p* ≤ .001; SDQ-DP 15-item: *r* = .68, *p* ≤ .001) and negatively associated with emotion regulation (SDQ-DP 5-item: *r* = − .30, *p* ≤ .001; SDQ-DP 15-item: *r* = − .42, *p* ≤ .001).

Both SDQ-DPs were significantly and negatively associated with academic performance (SDQ-DP 5-item: *r* = − .23, *p* ≤ .001; SDQ-DP 15-item: *r* = − .38, *p* ≤ .001) and student-teacher closeness (SDQ-DP 5-item: *r* = − .11, *p* = .028; SDQ-DP 15-item: *r* = − .15, *p* = .002). Only the SDQ-DP 15-item was significantly and negatively correlated with academic self-efficacy (*r* = − .12, *p* = .012) and the perception of a positive school climate (*r* = − .10, *p* = .036). Finally, both SDQ-DPs were positively associated with student-teacher conflict (SDQ-DP 5-item: *r* = .45, *p* ≤ .001; SDQ-DP 15-item: *r* = .46, *p* ≤ .001) and teacher-reported peer problems (SDQ-DP 5-item: *r* = .50, *p* ≤ .001; SDQ-DP 15-item: *r* = .54, *p* ≤ .001).

## Discussion

The co-occurrence of signs of emotional, cognitive, and behavioral difficulties in children and adolescents has been associated with a wealth of adverse outcomes in community and clinical samples [10–12]. This complex picture of symptoms is usually evaluated using a Dysregulation Profile, such as the SDQ-DP, defined with a 5-item or a 15-item scale [8, 9]. However, only a few studies evaluated its psychometric properties. The current study explored, for the first time, the structure and psychometric properties of the Italian version of the SDQ-DPs teacher-report (5-item and 15-item) and their associations with two constructs central to emotionality in youths (i.e., lability/negativity and emotion regulation) and different aspects relevant in the school context.

A first Confirmatory Factor Analysis of the SDQ-DP 5-item revealed poor goodness-of-fit. In order to find signs of model misspecification, modification indices have been explored, and they suggested a strong covariance (M.I. = 197.01) between the error terms of item 8 and item 13. Only after accounting for the errors covariance term, the CFA showed acceptable goodness-of-fit, partially supporting the single factor nature of the SDQ-DP 5-item teacher-report. Bentler and Chou [29] consider it not appropriate to force large error terms, such as those found in our sample, to be uncorrelated when dealing with real data. Indeed, as explained by Byrne [24] these error covariances represent systematic measurement errors that might be caused by the characteristics of the items themselves (e.g., a common source of error or some redundancy due to content overlap) and/or the respondents (e.g., item interpretation). In our case, both items belong to the same SDQ subscale assessing children’s emotional symptoms, and even though worded in different ways, their content might appear similar (“*Many worries*” and “*Often unhappy”*). Also, it is reasonable to hypothesize that children who are frequently worried might appear less happy or sad or perceived as such by teachers who would eventually interpret and rate those items similarly. Based on these theoretical aspects and Byrne’s [24] suggestions on post-hoc analyses in structural equation modeling, we consider the respecification of the initial model justified. However, it is necessary to highlight that modification indices in general, and so those that led to the respecification of the SDQ-DP 5-item model, are entirely determined by the data and thus might represent some idiosyncratic characteristics of our sample and not of the general population. As the current study is the first one exploring the factor structure of the SDQ-DP 5-item teacher-report, more studies are needed to better understand its structure. Overall, the results pertaining to the factor structure of the SDQ-DP 5-item teacher-report cannot be considered conclusive and must be interpreted with due caution.

In line with Deutz et al. [9], our results showed that a bifactor model best fitted the data related to the SDQ-DP 15-item. Even though increasingly used in psychology and psychopathology/psychiatry research, bifactor models have raised some concerns that are important to be aware of when interpreting the results of the current study. The main issue associated with bifactor models is their tendency to show superior goodness of fit in model comparison due to overfitting the data while capturing unwanted noise [30, 31]. Despite the concern raised by bifactor models, they are considered “useful for their ability to separate indicator variance associated with a general factor from variance associated with narrower group factors or specific indicators” [32] (p. 19). Also, they can provide information on whether a general factor (i.e., DP) is present, its strengths, and its content, highlighting the characteristics that better describe it [32].

Moreover, bifactor models are usually preferred to higher-order ones when the theoretical framework considers the specific factors not as components of the general factor but as separate factors. This might be particularly suitable when studying youth dysregulation since behavioral–genetic studies and theoretically based models showed that, when described by a DP, dysregulation is distinct from its specific components [33–36], and so it should be conceptualized as a transdiagnostic syndrome that exists next to specific problems (i.e., Hyperactivity–Inattention, Emotional Symptoms, Conduct Problems) [37]. A bifactor model would better capture this conceptualization, as also suggested by our results and several studies exploring the DP with different measures (e.g., SDQ, CBCL, Youth Self Report, Teacher Report Form) and samples [9, 36, 38, 39]. Finally, the examination of the factor loadings revealed that all the items significantly loaded into the general factor DP and their specific factors (see Table 4), with most of the factor loadings of the general factor above 0.40, supporting the bifactor nature of the SDQ-DP 15-item teacher-report.

Also about the factor loadings related to the general factor DP, overall, our findings showed that smaller weights were associated with items from the Emotional Symptoms scale, for instance, item 3 (“*Often complains of headaches”*), item 8 (“*Many worries*”), or item 24 (“*Many fears*”). This can suggest that externalizing symptoms (e.g., “*Restless, overactive*,” “*Easily distracted*,” “*Often fights*”*)* might better capture the manifestations of children’s dysregulation in the school context. At the same time, externalizing problems are easier to notice and identify, and teachers usually perceive them to be more severe and more concerning than internalizing problems [16]. Finally, it is noteworthy to mention that also item 22 (“*Steals*”), which is part of the Conduct Problems scale, had very low factors loadings. This result might be partially accounted for by the fact that stealing is probably not very common in young children drawn from the general (vs. clinical) population like those included in our sample.

The current study also aimed to test the measurement invariance across gender of the two versions of the SDQ-DP. Results showed full configural and metric invariance for the two scales, which means that the SDQ-DP, assessed with 5 or 15 items, has a similar structure and factor loadings for boys and girls, implying that the construct has the same significance for the two groups. However, we found evidence of full scalar invariance only for the SDQ-DP 15-items and not for the 5-item scale. This suggests that other factors (e.g., social norms, developmental differences) might influence how boys and girls behave and are rated and, consequently, the DP scores when assessed with only 5 items. We found that, in general, boys reported higher scores in items 2 (“*Restless, overactive*”) and 12 (“*Often fights*”), which refer to externalizing symptoms, commonly lower in females [30]. Non-invariance of the intercepts calls for greater caution when testing for gender differences using the SDQ-DP 5-items.

Consistent with other studies, we found weaker internal consistency and stability for the SDQ-DP 5-item than for the 15-item scale [8, 9]. This was expected as longer measures are usually more reliable than shorter ones. For this reason, we evaluated the SDQ-DPs internal consistency with mean inter-item correlations, which are less sensitive to measures length, and found that the items included in the SDQ-DP 5-item are correlated with each other and most likely evaluate a single construct. In addition, the SDQ-DPs were found reliable in a second assessment, indicating that dysregulation tends to remain stable, at least after six or twelve months.

Finally, this study aimed to provide initial evidence of the SDQ-DPs construct validity and explore their associations with different variables relevant in the school context. After controlling for gender, results showed that both the SDQ-DP versions were similarly associated with the subscales of the Emotion Regulation Checklist. Specifically, they were positively correlated with lability/negativity, which assesses inflexibility, lability, and dysregulated negative affect, and negatively correlated with emotion regulation, which measures proper emotional expression and emotional self-awareness.

Regardless of the SDQ-DP scale used, co-occurrence of emotional, cognitive, and behavioral dysregulation in students was associated with poorer school performance and greater relational difficulties with both teachers (low closeness and high conflict) and classmates. The SDQ-DP 15-item was also associated with lower academic self-efficacy and a worse perception of the school climate. These findings are in line with previous studies indicating that dysregulation can be related to youths’ psychosocial and academic functioning, as well as peer relationships [11–13]. At the same time, they shed light on associations between dysregulation and other aspects of youths’ school life, such as student-teacher relationships, academic self-efficacy, and perception of the school climate, not previously investigated.

These results need to be interpreted in light of several limitations. First, the construct validity of the SDQ-DPs has been tested only with the Emotion Regulation Checklist. Despite being a widely used and validated instrument, it focuses mainly on emotion regulation and does not include the evaluation of dysregulation at the behavioral and cognitive levels. Moreover, we did not include other DP measures (e.g., CBCL-DP), which would have provided a more thorough validation of the SDQ-DP. In addition, both the SDQ and ERC were assessed by teachers, which could account for shared variance. Also, peer problems were assessed with the same questionnaire used for the DPs. Although a strength of the study is the employment of self-report and teacher-report instruments, future studies should employ a broader range of measures to validate the SDQ-DPs and explore their correlates. Even though the CFA was conducted on a large (*N* = 1000) sample, the measures used to test the construct validity and outcomes in the school context were not available for all the students, reducing these analyses’ power. Finally, future studies should investigate the longitudinal validity of the SDQ-DPs, as well as their reliability after a longer period.

## Conclusion

Co-occurring emotional, cognitive, and behavioral dysregulation is a serious issue that affects a considerable number of children and adolescents, causing severe adjustment problems. Teachers have a privileged point of view on youths’ lives and can play an essential role in recognizing students with difficulties. Moreover, schools guarantee easy access to a significant number of youths simultaneously, making them precious for screening campaigns led by mental health professionals. These aspects highlight the need for reliable teacher-report measures that would facilitate the identification of students at greater risk for adverse outcomes and, consequently, the implementation of preventive and targeted interventions. Within this context, the current study evaluated the psychometric properties of the SDQ-DPs 5-item and 15-item teacher-report, showing that they could serve this scope but also provided evidence for more informed use of the two scales. The SDQ-DP 15-item showed higher internal and test-retest reliability, making it somehow more recommended (see also [9]). However, results also endorse a cautious use of the 5-item scale, which might be particularly parsimonious and useful in settings characterized by tight time constraints.

## Summary

The presence of emotional, cognitive, and behavioral dysregulation during childhood and adolescence has been recognized as a significant risk factor for several outcomes, including internalizing and externalizing problems, difficulties in the school context, and might also impact youths’ psychosocial functioning and relationships with their peers. This evidence highlighted the need for reliable measures to assess it, such as the Strengths and Difficulties Questionnaire Dysregulation Profile (SDQ-DP), defined by 5 or 15 items. The current study explored for the first time the factor structure, internal consistency, and test-retest reliability of the Italian version of the SDQ-DPs teacher-report in a large sample of children (*N* = 1000; age 7–12 years). The gender invariance of the SDQ-DPs, construct validity, and associations with school variables were also evaluated. Confirmatory factor analyses showed that a first-order model for the SDQ-DP 5-item and a bifactor model for the SDQ-DP 15-item best fitted the data, with results being more defined for the SDQ-DP 15-item than the 5-item scale. Full measurement invariance across gender was confirmed only for the 15-item scale. Internal and test-retest reliabilities were weaker for the 5-item scale. Both SDQ-DPs were similarly associated with a measure of emotion regulation skills and some school variables, including student-teacher relationships, academic self-efficacy, and perception of the school climate. Overall, these findings provide indications for a more conscious and informed use of the SDQ-DPs 5-item and 15-item.

## Declarations

### Note

DP: Dysregulation Profile.
